# Metastatic Progression of Human Melanoma

**DOI:** 10.3390/cancers15041225

**Published:** 2023-02-15

**Authors:** József Tímár, Andrea Ladányi

**Affiliations:** 1 Department of Pathology, Forensic and Insurance Medicine, Semmelweis University, H-1191 Budapest, Hungary; 2 Department of Surgical and Molecular Pathology and the National Tumor Biology Laboratory, National Institute of Oncology, H-1122 Budapest, Hungary

This Topical Collection, comprising 13 papers (10 original articles and 3 reviews), addresses various aspects of the field of melanoma progression: genomic and proteomic approaches, experimental studies, the questions of sentinel lymph node dissection, and metastasis formation of uveal and conjunctival melanomas is also discussed.

The review of Cherepakhin et al. [1] summarizes the genomic and transcriptomic features of melanoma genesis and progression. Our knowledge on cutaneous melanoma genomics mostly derives from primary tumors, local recurrences and metastatic lymph nodes. The driver genes are clearly identified, such as BRAF, NRAS, NF1 and KIT. Meanwhile, the genomics of rare histological variants, acrolentiginous, mucosal or uveal, are quite different concerning both driver oncogenes and suppressor genes. In The Cancer Genome Atlas (TCGA), therefore, a very limited number of data is present concerning the genomic progression of cutaneous melanoma and even much less concerning the rare histological variants. The sex-dependent progression of melanoma is a well-known feature, and the recent discovery of the loss of the DDX3X gene on the X chromosome during progression may shed some light on this biological phenomenon. Most of the data on metastasis genes of melanoma derive from primary tumors with different prognosis and revealed GULP1, DAB2, P4HA2 and KDELR3 as markers. Other studies identified transcription factors promoting progression: SMAD7, ZEB/AXL complexes, SOX2-GLI1 or YAP-TAZ-PAX3 [1].

A genomic study on copy number variations (CNVs) of visceral metastases of human melanoma by Papp et al. [2] revealed that the progression to key sites such as brain, lung and liver is associated with organ-specific features. Copy number gains (CNGs) characterized lung and liver metastases while copy number losses (CNLs) were unique to brain metastases. Furthermore, those CNLs were found to affect DNA repair genes, suggesting an organ-specific genetic vulnerability. It is of note that HGF/CMET co-amplification was identified as another molecular feature of brain (and lung) metastases. On the other hand, development of lung metastases was associated with the amplification of several immune cell genes, the majority of which is translated to protein overexpression and termed here as immunogenic mimicry, a clear indication of a novel immune escape mechanism. BRAF mutation and amplification were also molecular features of lung metastases [2].

An interesting experimental study shed some light on the contribution of host cells in the development of melanoma lung metastasis. Dacheaux et al. [3] constructed a mouse model where the ATX-producing capability of alveolar epithelial cells was knocked out. In such a model the lung metastatic ability of B16-F10 melanoma cells was significantly reduced, proving that the host derived autocrine/paracrine motility factor, ATX, is a significant contributor to melanoma lung metastasis formation.

In this Topical Collection, several papers deal with the proteome of cutaneous melanoma. Gil et al. [4] analyzed a small primary melanoma cohort and revealed the importance of mitochondrial activation in progression. Moreover, they detected a sex-dependent profile: in women MHC-related proteins as well as PSMB8 were upregulated, while in men Ca-binding proteins were detected. It is of note that in women’s melanoma, acetylation of several proteins was predominant, while in men’s melanoma, phosphorylation of chromatin modulators and some kinases such as MAPK1 and CSNK2A2 were characteristic. Almeida et al. [5] analyzed plasma proteome of melanoma patients with a novel high sensitivity mass spectrometry (MS) technology. They were able to detect several known or putative melanoma markers in the plasma, including the well-known LDH, but also CD44, MCAM, and several extracellular matrix proteins such as osteopontin, laminin, tenascin C, collagens, C-reactive protein and serum amyloid A. Beside them, more than 660 various proteins have been detected in melanoma patients. Szadai et al. [6] performed proteome analysis on 90 FFPE melanoma samples and selected those proteins that have prognostic and/or predictive significance. They have identified TRAF6, ARMC10, CDK2, ITGA5, CAMK4, and WIPI1 as negative prognosticators and AIF1 and PPIF as positive ones. In the immunotherapy subgroup, ARGN and ICAM2, among others, were identified as positive predictors while the VEGFA-VEGFR pathway as negative predictors. In the target therapy subgroup, PAK4, MAP2K2, PTEN, and DEPTOR have been identified as resistance factors. Zhang et al. [7] looked at other prognostic factors of metastatic melanoma at the primary tumor level and identified acetylated DNMT1 as a positive prognostic factor along with TIP60 and USP7.

In relation to target therapy resistance (BRAFi+MEKi), a nice experimental analysis was reported in human melanoma cell lines from Patel et al. [8]. A gene expression profile characteristic of target therapy resistance was identified, revealing 1591 differentially expressed genes in resistant cell lines. RT-PCR confirmed upregulation of CXCL12, COL5A1, ABCC3 and CHST15 and downregulation of DMRT2, MRGPRX4 and VEPH1 genes. At proteome level, resistant cells were shown to overexpress CapG, enolase-2, galectin-3, osteopontin, and survivin as potential resistance markers [8].

Intermittent hypoxia (IH) may provide a microenvironmental factor for the progression of skin melanoma. In the study of Khalyfa et al. [9], exosomes have been derived from obstructive sleep apnea (ASA) patients and intermittent hypoxia was applied to melanoma cell lines characterized by different mutation status. Patient-derived exosomes and IH stimulated cell proliferation and migration of BRAF mutant cells when STK11 mutation is co-occurring. Furthermore, tumor cell-derived exosomes upon IH stimulated CXCL10 and IL6 production of macrophages. These data support the notion that IH can affect biological behavior of melanoma cells which depends on their unique genetic makeup.

Sentinel lymph node (SLN) testing is a standard of cutaneous melanoma management and positivity indicated complete lymph node dissection (CLND) for decades. This practice was based on the presumption that the SLN of melanoma is a source of distant dissemination. However, recently this practice was questioned, demonstrating that CLND following positive SLN dissection result did not affect survival. In the study by Susok et al. [10], a retrospective analysis of the long-term survival of cutaneous melanoma patients was performed, which indicated that at 10 and 20 years there is no difference in survival of melanoma patients who underwent CLND or not, further supporting this new practice. Another large study by Liszkay et al. [11] analyzed the association of BRAF and NRAS mutations and other known prognostic factors with SLN status. According to the results, only the thickness of the primary tumor predicted SLN positivity. On the other hand, the study indicated that NRAS mutant status is a poor prognostic factor in terms of progression-free survival, independently of SLN positivity, indirectly supporting again the theory that the SLN is not the source of visceral progression of melanoma.

Melanoma can derive not only from the skin but from mucosal surfaces or retina as well. Conjunctival melanoma is a relatively rare form of melanoma with massive metastatic potential. Genetically it is close to the cutaneous form, carrying UV mutational signature. Furthermore, the driver oncogene pattern is also similar to the cutaneous form with BRAF, NRAS, NF1 and KIT mutations. Conjunctival melanoma is associated with prominent lymphangiogenetic potential but also with frequent local recurrence or distant metastasis formation. Its clinical management was based on radical surgery, frequently resulting in orbital exenteration. However, this situation is changing, since radical surgery of the primary tumor did not result in significant improvement of survival and metastatic progression. This situation is similar to the CLND of cutaneous melanoma, since both melanomas are characterized by high aggressiveness and early dissemination potential. New surgical and irradiation modalities are nicely summarized in the review of Nahon-Estève et al. [12].

Uveal melanoma (UM), derived from the retinal pigment cells, is another rare form of melanoma which is not induced by UV. A nice comprehensive review of Rossi et al. [13] summarized our recent knowledge on the molecular genetics and biology of this unique form of melanoma. The main signaling pathway which is affected is the G protein coupled pathways affecting not only the MAP but also the YAP/TAZ signaling. The constitutive activity of this signaling pathway is due to mutations of GNAQ and GNA11 genes, but can also be due, although less frequently, to CYSLTR2 and PLCB4. Uveal melanomas are characterized by a very low tumor mutational burden (TMB), thus by a low number of genetic alterations. During genetic and biological progression, BAP1 and SF3B1 mutations develop. It seems that the progression of UM is dependent on the histone H2a posttranslational modification induced by BAP1/EZH2 and PCR1. Due to the immune privilege of the eye, UM is unique immunologically. There is no lymphatics, there is a blood-retina barrier, the vitreal fluid is rich in immunosuppressive cytokines such as TGF-β, MIF and FAS ligand. On the other hand, visceral metastatization of UM occurs almost exclusively to the liver. Although the molecular markers of such dissemination have been known for some time (a MET mediated process), specific inhibitors were clinically ineffective in controlling it. It seems that the unique immune escape mechanisms of UM developed in the eye somehow promote metastasis development in the liver as well. These unique immunological mechanisms may offer novel forms of immunotherapy of UM [13].

In conclusion, this Topical Collection presents recent data covering several aspects of metastasis formation of melanoma, including genomic, proteomic, and experimental studies, among others, which may provide novel therapeutic targets as well as prognostic factors for the management of metastatic melanoma.
